# Design, simulation and experimental analysis of a monolithic bending section for enhanced maneuverability of single use laparoscopic devices

**DOI:** 10.1038/s41598-024-53914-3

**Published:** 2024-02-09

**Authors:** Kai Uhlig, Sascha Bruk, Matthieu Fischer, Konrad Henkel, Franz Brinkmann, René Körbitz, Ronny Hüttner, Malte Pietsch, Phillip Hempel, Axel Spickenheuer, Markus Stommel, Andreas Richter, Jochen Hampe

**Affiliations:** 1 Leibniz Institute of Polymer Research Dresden, Institute of Polymer Materials, 01069 Dresden, Germany; 2 Chair of Microsystems, Dresden University of Technology, 01187 Dresden, Germany; 3 Department of Medicine I, Dresden University of Technology, University Hospital Dresden, 01307 Dresden, Germany; 4 Else Kröner-Fresenius Center for Digital Health, Dresden University of Technology, 01307 Dresden, Germany; 5 Admedes GmbH, Pforzheim, Germany; 6 Chair of Polymer Materials, Dresden University of Technology, 01069 Dresden, Germany

**Keywords:** Flexure hinge, Bending section, Nitinol, Laparoscopes, Digital image correlation, Laser based processing, Gastroenterology, Engineering, Materials science

## Abstract

Standard laparoscopes, which are widely used in minimally invasive surgery, have significant handling limitations due to their rigid design. This paper presents an approach for a bending section for laparoscopes based on a standard semi-finished tube made of Nitinol with laser-cut flexure hinges. Flexure hinges simply created from a semi-finished product are a key element for realizing low-cost compliant structures with minimal design space. Superelastic materials such as Nitinol allow the reversible strain required for this purpose while maintaining sufficient strength in abuse load cases. This paper focuses on the development of a bending section for single use laparoscopic devices (OD 10 mm) with a bending angle of 100°, which enables the application of 100 µm diameter Nitinol actuator wires. For this purpose, constructive measures to realise a required bending curvature and Finite Element Analysis for determining the strain distribution in the flexural region are applied and described for the design of the flexure hinges. In parallel, the influence of the laser-based manufacturing process on the microstructure is investigated and evaluated using micrographs. The deformation behavior of the bending section is experimentally determined using Digital Image Correlation. The required actuation forces and the failure load of the monolithic bending section is measured and compared to a state of the art riveted bending section made of stainless steel. With the developed monolothic bending section the actuation force could be reduced by 50% and the available inner diameter could be increased by 10% while avoiding the need of any assembly step.

## Introduction

Minimally invasive endoscopic procedures have revolutionized modern medicine^1^. For instance, with over 6 million gastrointestinal endoscopies per year in Germany, they also play a significant role in quantitative terms. The complex and expensive devices used are currently reused several times, which means that despite extensive cleaning, infection problems can occur^2^. To eliminate these disadvantages, there is currently a trend towards the development of single use instruments (SUI). SUI requires simple and cost effective, easy to manufacture and easy to mount functional components. This paper addresses the bending section which is required in those devices.

**Figure 1:**
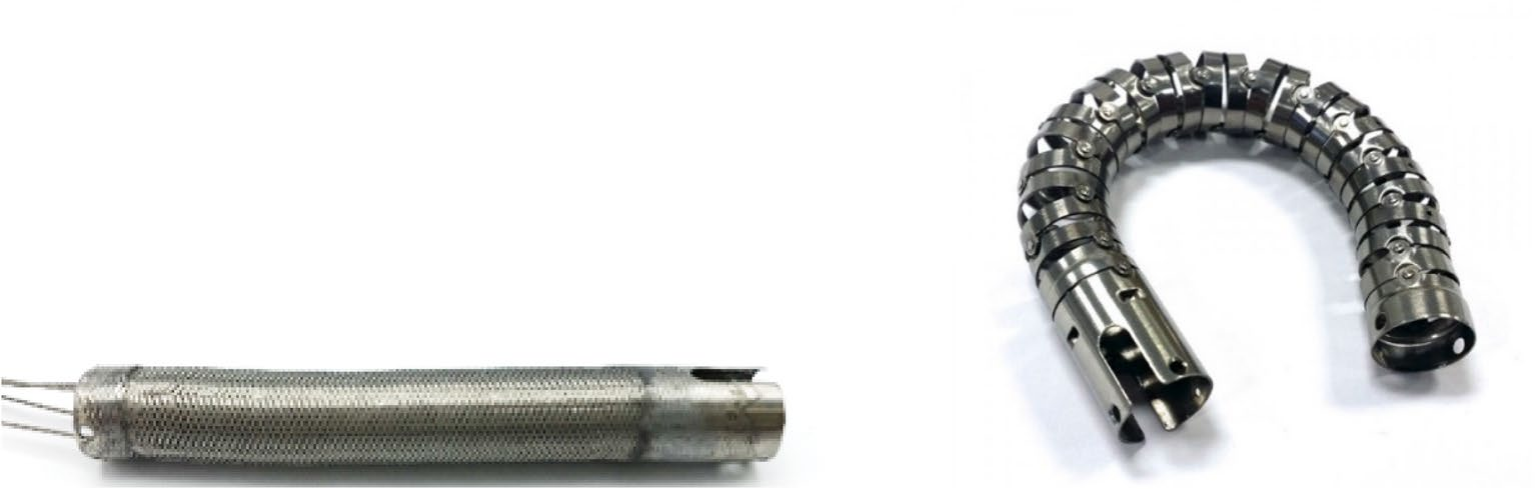
Example of a bending section in a typical state of the art endoscope—with welded mesh and mounted bowden wires (left) and the pure inner bending section (right, device class OD 10 mm), Source Endoscopeparts.com®.

To date in current endoscopes (device class outer diameter (OD) 10 mm) joints of the bending section are made of a combination of several cylindrical individual segments which are connected by rivets or cylindrically shaped lugs to form a system of sliding hinge joints. They are often made of a biocompatible stainless steel. The individual joints are alternately rotated 90° around the central axis so that the resulting framework is free to bend in all directions. The guide for the bowden cables used to actuate the endoscope is also integrated into the joint. All working channels, data and supply lines of the endoscope run through the inside of the framework. From the outside, the joint is additionally supported by a wire mesh, as shown in Fig. 1.

The disadvantages of this design are the large number of individual parts, the resulting assembly effort and consequently high manufacturing costs. Furthermore, the mechanical axial and rotational backlash in the riveted joints of the bending section lead to an insufficient steering precision of these devices. In addition, such bending sections do not have a defined zero position due to their inherent design, which means that the joint does not return to the zero degree position by itself.

With a flexure hinge approach all these disadvantages might be addressed. Those joints do not require assembly, exhibit no friction, wear or backlash and can be produced via laser-based techniques on semifinished products. The elimination of assembly steps and immediate readiness for use make this design particularly interesting for price-optimized disposable instruments.

Several research efforts have been conducted in the field of flexural hinges for endoscopic surgery devices. In^3^, Howel presented a wide variety of approaches for the design of compliant mechanisms in a general context. Coemert et al.^4^ developed a design optimization method for complied structures based on flexural hinges to achieve a homogeneous stress distribution and thus a longer fatigue life. However the approach does not cover superelastic materials like Nitinol. A flexure hinge manipulator with two subsequent bending sections with one degree of freedom (DOF) based on a segmented profile with a rectangular cross-section made of Nitinol by electrical discharge machining (EDM) for frontal sinus operations was demonstrated in^5^. The subsequent bending sections are designed specifically for the anatomical requirements of the frontal sinus and cannot compare with requested flexibility of modern endoscopes which includes a 3D spherical range of motion and bending angles of at least ± 90°. A manipulator for hip revision surgery made of two nested Nitinol tubes that are segmented by EDM process is described in^6^ having a maximum outer diameter of 6 mm and a 4 mm wide inner lumen, allowing bending in one plane. Having a sufficient bending angle in that plane, this design does not allow for active deformation in another direction and has to be rotated around the insertion axis to move its tip out of plane. Both studies have in common that Nitinol was selected as the construction material. Conventional biocompatible high strength materials such as stainless steel do not provide enough reversible strain to achieve the required articulation angles.

Nitinol is an intermetallic alloy of nickel (Ni) and titanium (Ti) developed at the Naval Ordnance Lab in the 1960s^7^, where the two elements are present in approximately equal atomic proportions. Nitinol exhibit two different crystal phases. The interpenetrating simple cubic structure referred to as austenite is stable at higher temperatures and the monoclinic crystal structure referred to as martensite is stable at lower temperatures. Additionally, in the martensite crystal structure a reversible solid-state phase transformation between two different martensite crystal phases is possible, known as twinned and detwinned martensite^8^. The specific structure of Nitinol enables shape memory properties below and superelastic properties above the austenite–martensite transformation temperature^9^. Due to the thermal hysteresis Nitinol exhibit four transition temperatures related to the transformations of austenite-to-martensite and martensite-to-austenite. Starting from full austenite, martensite begins to form as the Nitinol is cooled down to the so-called martensite start temperature (Ms). The temperature at which the transformation is complete is called the martensite finish temperature (Mf). When the Nitinol is in its full martensite state (below Mf) and is subjected to heating, austenite starts to form at the austenite start temperature, As, and finishes at the austenite finish temperature, Af.

For the design of flexure hinges superelastic material properties are preferable. To enable super elastic properties, operating temperature above the austenite transformation finish temperature of the material are necessary. When deforming a Nitinol structure in its austenite phase, at a certain stress level, martensite is formed from the austenite phase while simultaneously changing structural shape. This effect enables up to approximately 6–8% reversible strain and makes this material particulary well suited for flexure hinges. The temperature above which the austenitic phase is present in Nitinol can be set within certain limits by adjusting the composition of Ni and Ti^10^. The required stress level for the austenit-martensit transformation increases with higher temperature and the effect sustains only at temperatures between the austenite finish temperature and a higher critical temperature^11,12^. Nitinol is therefore only suitable for flexural hinges which have an operating temperature in a certain temperature range. Regarding its suitability for medical application, several in vitro and in vivo experiments have demonstrated the biocompatibility of Nitinol^13–15^ and already many medical devices and implants make use of this material^16,17^.

For modeling the mechanical behavior of Nitinol in its superelastic state, from tension-based one-dimensional phenomenological descriptions^18–20^ up to entirely three-dimensional characterizations^21–24^, constitutive models have been developed in the last decades. These approaches were integrated into commercially available finite element software. To this end, ANSYS® and ABAQUS® use the modeling approach developed by Auricchio et al. to describe the superelastic behavior of Nitinol^22,25,26^.

Due to its high hardness and the thermal sensitivity of the microstruture, mechanical processing without affecting the mechanical properties of Nitinol is challenging^27^. Laser technology provides the required high precision and processing speeds for the processing of Nitinol structures based on semifinished products, when the heat management is controlled. Especially ultra-short pulsed lasers (picosecond and femtosecond lasers) enhances laser energy coupling through a nonlinear multi-photon absorption mechanism and produces virtually no residual heat^28^. With ultra-short pulsed lasers the microstructure and thus the mechanical properties can be preserved without any heat affected zone (HAZ) in the processed material^29,30^.

Regarding the actuation of endoscopic bending sections, Nitinol is considered as actuator material using the shape memory effect. Therefore Nitinol-wires, coils or spring-elements are used. In^31^ independent Nitinol actuators with spring-like geometry are used in a multi-segmented design with 3 sections of 3 actuators. The actuators double in length when heated, thereby deflecting the segment guided by a superelastic bias-spring. The thin geometry however, makes this design prone to kinking under axial or lateral loading. Two approaches using Nitinol actuators to achieve bending of articulated endoscopes are presented in^32^. First, by using a commercially available endoscope head with two pairs of antagonistic Nitinol wires attached to cables running through the head, a 2 DOF bending was achieved, reaching about 100° maximal bending angle. Second, using specifically designed Nitinol plate springs alternating 90° between each ring segment, independent DOF for each segments could be achieved with a bending angle of 10.5° for one segment. While demonstrating good performance of the Nitinol-actuators, however, in both designs the bending mechanisms still require many assebly steps with the plate spring approach furthermore requiering additional measures for electrical insulation. A design using two antagonistic Nitinol-actuator blades with 30° curvature between two ring segments with integrated thermoelectric cooling is presented in^33^ with diameter of 4 mm and 9 mm of length. Allowing only one blade to be activated at the time, deflection up to 20° in one direction is possible, making this system small and actively coolable but complicated with regrads to assembly and controlling.

In this paper, we propose a 2 DOF laser cut monolithic bending section using superelastic Nitinol with tendon-based activation for the application in single use laparoscopic devices, initially focusing on the use as a flexible imaging instrument equipped with a camera tip. The proposed manipulator is characterzied by a defined “zero” position and low operating forces while maintaining a high torsional rigidity for precise operation.

For the addressed use case, Nitinol in its superelastic configuration has the ability to repeatedly withstand the required large deformations while maintaining a sufficient strength^9^.

## Materials and methods

### Design of a 2 DOF monolithic Nitinol bending section

Regarding the design of the novel flexible tip for laparoscopic devices several initial operating conditions were defined in advance. For reasons of practical relevance, a device outer diameter of 10 mm was selected, matching that of common rigid laparoscopes. As actuation, a tendon-based approach of two pairs of antagonistic tensile preloaded Nitinol actuator wires with a diameter of 100 µm is assumed, supporting the requirement for a large diameter of the internal lumen. Considering the operating range of bending sections in state of the art endoscopic devices (OD 10 mm) the length of the compliant zone is set to 64 mm with two DOFs and a total bending angle in one direction of 100°.

**Figure 2:**
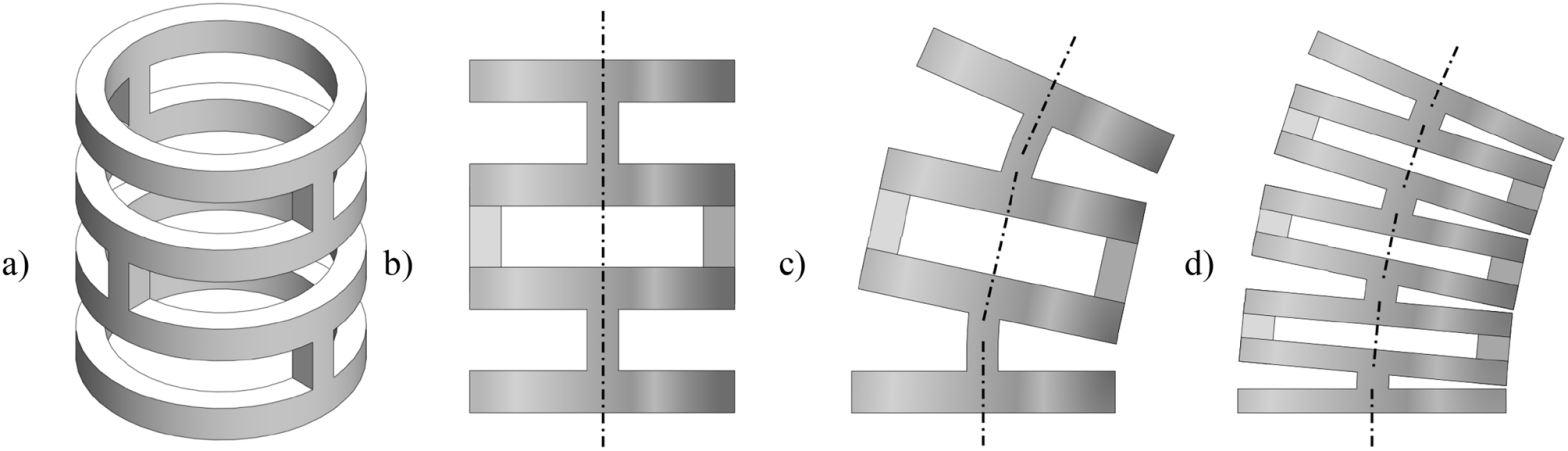
Design principle of the a monolithic 2 DOFs bending section based on the tubular base geometry with alternating cuts showing (**a**) an isometric view of the bending section, (**b**) side view of the undeformed section with two flexural hinges for in-plane bending, (**c**) side view of the deformed section with two hinges, and (**d**) side view of a deformed bending section with four flexural hinges for in-plane bending, showing a closer approximation to a circular arc line.

Due to the preloaded actuator wires, the bending section is under constant compressive stress. Under this loading condition, the structure is supposed to not deform more than approximately 1 mm in axial direction to provide a sufficient rigidity for precise steering and to achieve the required bending angle. Thus, flexure hinge designs as described in^4^ are not suitable for this loading condition due to their comparatively high axial compliance. As base material for the bending section a cylindrical tube made of Nitinol with an austenite finish temperature A_F_ of approx. 18°C obtained from Euroflex GmbH, with OD 10 mm and a wall thickness of 0.533 mm is used. The applied design principle to generate a monolithic 2 DOFs bending section is a symmetrically arrangement of orthogonally altering flexure hinges within the tubular base geometry (Fig. 2). In order to achieve 100° of bending within a section of 64 mm length and 10 mm of diameter a total shortening of 8.7 mm in arc length at the inner radius of the bending section is needed.

To obtain an almost circular path the number of segments should be as high as possible. However, in order to maintain axial stiffness, a certain height $d$ of the remaining material between the hinges is required.

**Figure 3:**
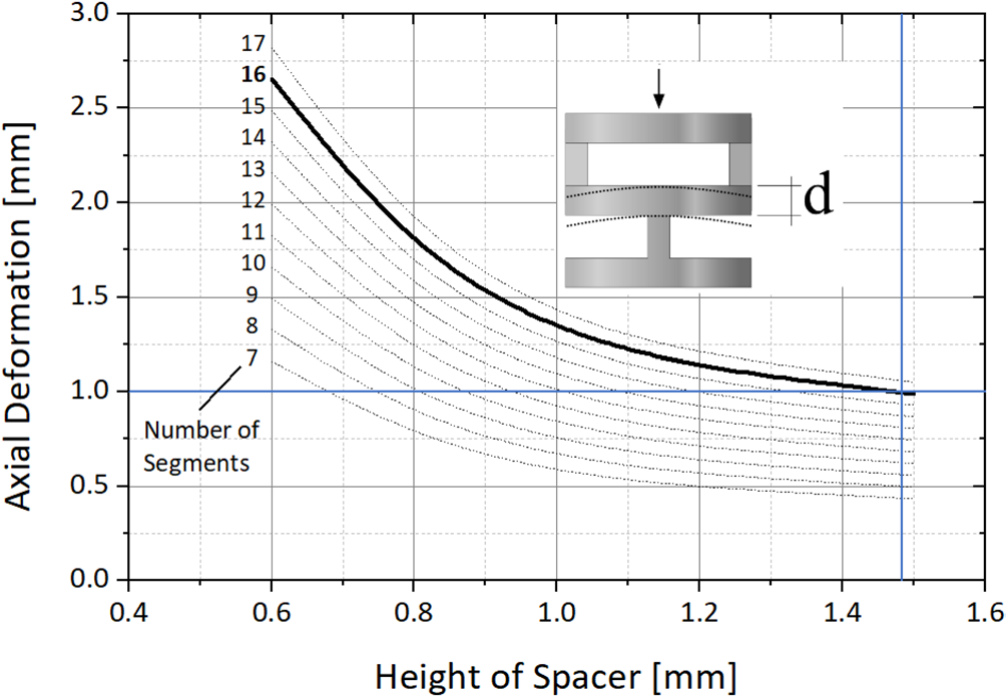
Finite element analysis (FEA) based calculation of the axial compression depending on the material height ${\varvec{d}}$ of the cylindrical sections between the hinges and the number of the sections.

Finite element method (FEM) was used to determine the necessary height of the cylindrical sections between the alternating bending hinges at the maximum number of hinge areas, so that an approximately circular bending can be achieved and the maximum compression of the entire bending section under defined tension of the actuating wires is not exceeded. To simulate the joint’s behavior, a half-model of the complete joint with symmetric boundary conditions is used for general deformation analysis. A detailed model of two hinge segments is used to consider more accurate deformation and resulting strains and to extrapolate the results to the entire structure. A number of 16 bending segments for each DOF, giving a total number of 32 hinge areas, and a height of 1.47 mm for the cylindrical sections, is identified as optimum over the 64 mm of working length (Fig. 3). Each hinge has a bending angle of 6.25° and a cut-out height *h* of 0.55 mm, which provides a 36.6 mm bending radius (inner radius 31.6 mm) of the entire bending section. The design shown in Fig. 1, on the other hand, has a bending radius of around 26 mm. The cylindrical sections requires a sufficient height to resist possible deformation due to the axial preload force, resulting in larger bending radii compared to the reference shown in Fig. 1. If no axial preload force were to act on the segments, a radius similar to that of the riveted reference design could be realized.

**Figure 4:**
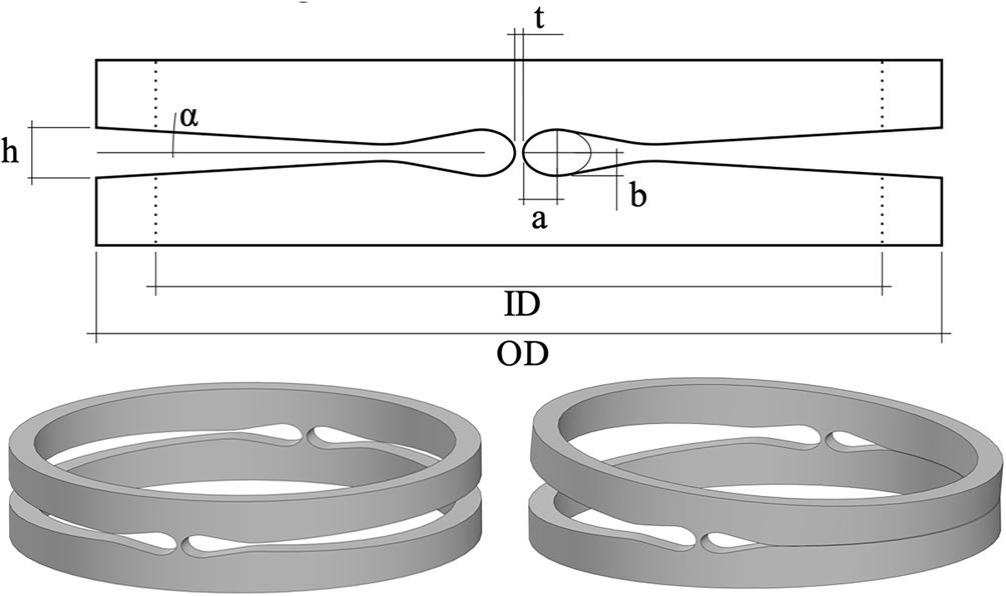
Relevant parameter of the hinge geometry (top) and single hinge segment in undeformed (left) and deformed state (right).

In order to reduce stress concentrations while still providing a high torsional stiffness and a precise deformation in the hinge area, an elliptic contour is chosen and further investigated. The ellipse ratio *k* is the ratio of the horizontal semi-axis *a* to the vertical semi-axis *b* to of the elliptical cut-out as shown in Fig. 4.

The contour of the cut-out forming the hinge area is specified, using the detailed FEA model of the unit cell with variable geometries to define the minimum thickness *t* of the hinge area and the ellipse ratio *k*. In order not to weaken the spacer area between two hinge sections and thereby lose compressive stiffness, the vertical axis of the ellipse is set equal to the cut-out height *h* of 0.55 mm. Base on the material data provided by Admedes GmbH a maximum allowable strain of 5.3% was defined for the joint area to assume an acceptable number of cycles for the hinge. Parameters at 100 µm thickness *t* and an ellipse ratio *k* of 1.6 are determined to be an optimum balance to reduce shifting of the virtual pivot point on the one hand and to allow for tolerable stress distribution in the hinge area on the other hand. In order to be able to limit the bending angle in a defined manner, the cutting contour in the opening area replicates the determined bending angle so that a surface contact between the opposing cut-out-surfaces occurs during deformation (see Fig. 4).

### FEA of the bending zone in the 2 DOF monolithic Nitinol bending section

**Figure 5:**
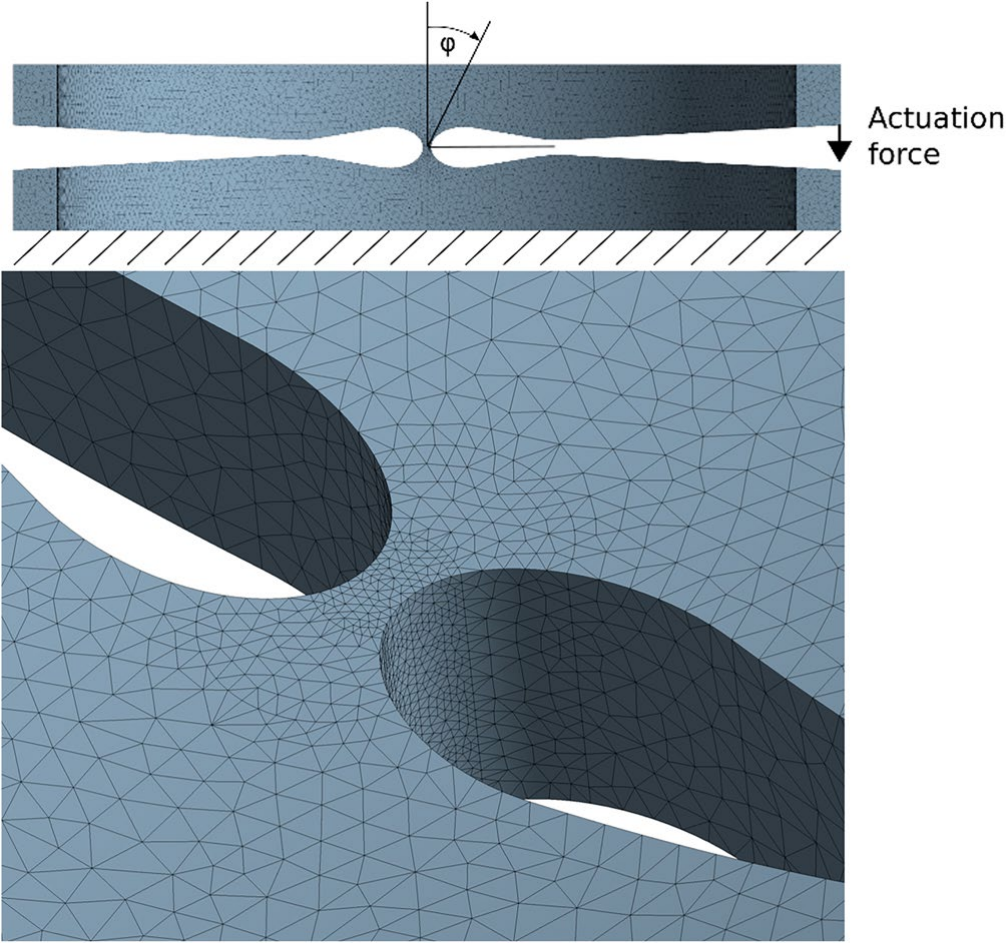
FEA half symmetry model, with boundary conditions and detail view of the mesh.

For the analysis of the strain distribution in the bending zone and to determine the required forces to fully articulate the tip, a single bending segement was investigated in detail using FEA. Due to the chosen design approach, the maximum deflection of the joint is geometrically limited. The finite element model was built using ANSYS Workbench 19. Due to the symmetric geometry and symmetric loading conditions, only a half model of the actual structure is simulated to reduce calculation time (Fig. 5). As element type a higher order 3-D 10-node tetrahedral structural solid element (Solid187) is chosen. The element size is set to 25 µm in the joint zone and gradually increases up to 200 µm in the periphery. The model is fully actuated by applying a force of 0.41 N at the upper edge of the structure. Since it is a half model this corresponds to 0.82 N for one complete section. The force value is equivalent to the actuation force of the used actuator elements. The nodes on the bottom area of the model are set to fixed support and the nodes of the mirror surface (i.e. the half models cut-surface) are constrained in the direction leaving the symmetry plane.

For the material model implemented in ANSYS® to simulate the superelastic effect (SE) of Nitinol, a total of eight material parameters are required (Young’s modulus of the austenite phase (E_A_), Poisson’s ratio (v), the material response ratio between tension and compression (α), the maximum residual strain (ɛ_r_), the starting stress value for the forward phase transformation (σ_SAS_), the final stress value for the forward phase transformation (σ_SAF_), the starting stress value for the reverse phase transformation (σ_SSA_) and the final stress value for the reverse phase transformation (σ_FSA_))^25^. The used material properties are specified in Table 1. Due to the fact that the testing of the structure is performed at room temperature (25 °C) and the intended operation temperature is 37 °C, material data for both temperatures are listed.

**Table 1:** Nitinol material data used for FEA.

Nitinol model parameters	Values	
Temperature	37 °C	25 °C
Young’s modulus of the austenite phase (E_A_)	73,889 MPa	73,889 MPa
Young’s modulus of the martensit phase (E_M_)	22,566 MPa	22,566 MPa
Poisson’s ratio (v)	0.3	0.3
Maximum residual strain (ɛ_r_)	0.053	0.053
Material response ratio between tension and compression (α)	0.2	0.2
Starting stress value for the forward phase transformation (σ_SAS_)	398 MPa	356 MPa
Finish stress value for the forward phase transformation (σ_SAF_)	427 MPa	382 MPa
Starting stress value for the reverse transformation (σ_SSA_)	210 MPa	188 MPa
Finish stress value for the reverse transformation (σ_FSA_)	151 MPa	135 MPa

**Figure 6:**
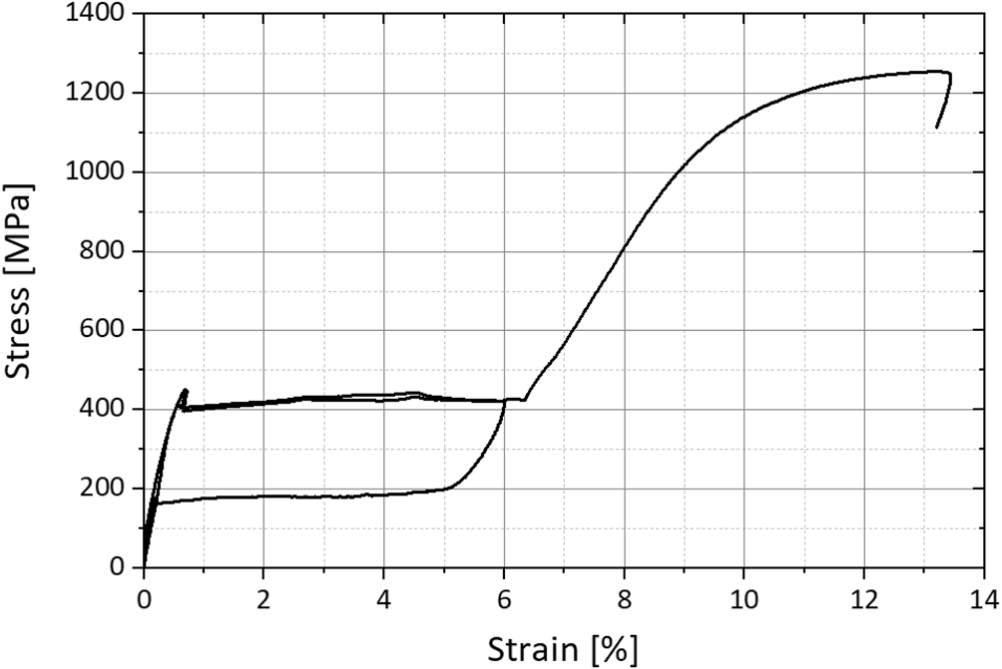
Stress strain behavior of the used Nitinol alloy at a Temperature of 37 °C (experimental data provided by Admedes GmbH).

These data were determined experimentally at Admedes GmbH on a material sample of the used tube provided by Euroflex GmbH. The stress–strain behavior of the used Nitinol alloy at a temperature of 37 °C is shown in Fig. 6.

**Figure 7:**
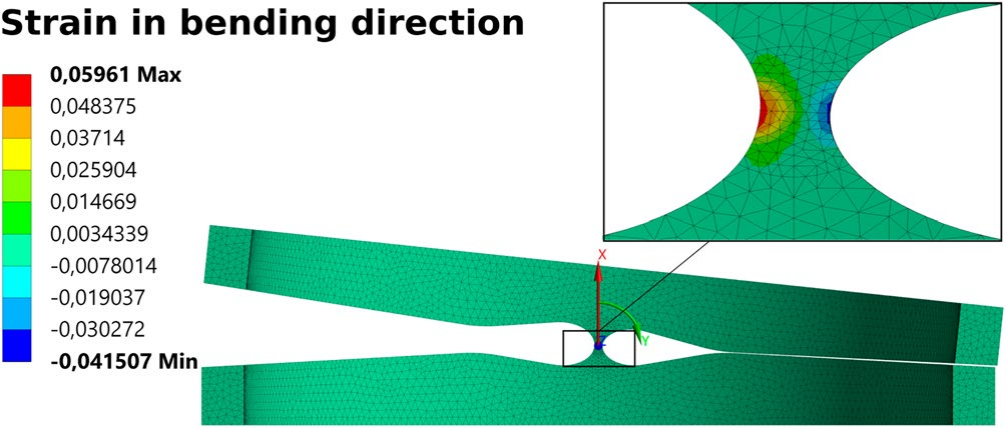
FEA result—strain in bending direction.

However, it has to be taken into account that the hysteresis loops in tension and compression are different^34^. In ANSYS®, these differences are characterized by the parameter α, which accounts only for the difference in stress at the starting stress value for the forward phase transformation. At the predefined hinge geometry (joint thickness of 100 µm and an ellipse ratio *k* of 1.6) the actuator forces are sufficient to fully deflect the hinge and the predefined strain limit of 6% is not exceeded. In Fig. 7 the strain distribution of one hinge element is displayed.

### Manufacturing of the monolithic nitinol bending section

**Figure 8:**
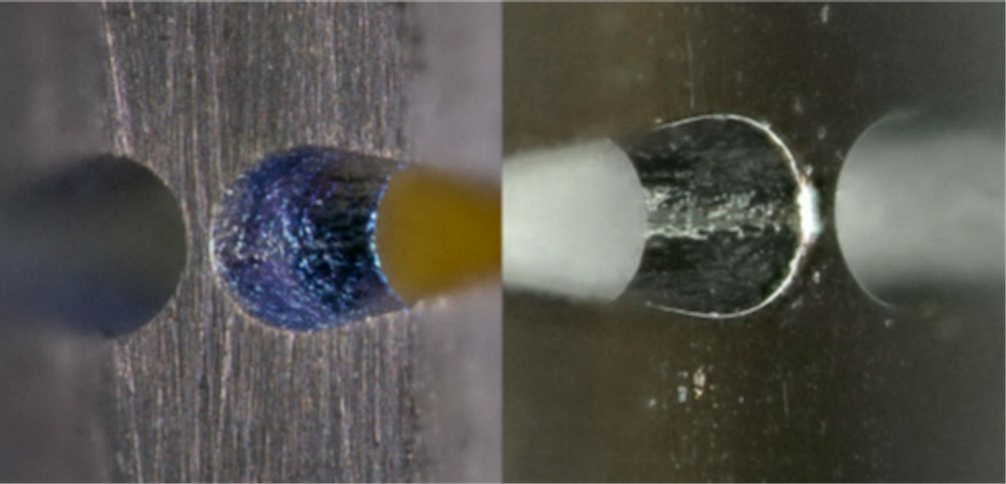
Surface quality of the Nitinol tube after laser cutting (left) and subsequent electropolishing (right).

The cut-outs in the hinge area of the Nitinol tube are made by means of a tube laser cutting machine. To avoid the problem of temperature-induced stress cracking and weld spatter due to the locally concentrated heat input during laser cutting, an ultra-short pulsed laser (femtosecond laser) was used to cut the joint geometry. The cutting was done at Admedes GmbH. Both, the starting material and the resulting surfaces after laser cutting show a rough surface, which makes the joint susceptible to premature failure as a result of the increased notch stresses. In order to smooth the surfaces after the cutting step, the joint was subjected to an electropolishing process which visibly smoothens the surface. Therefore, all cuts are set with an offset to the nominal dimension and then subjected to electropolishing, having the benefit of reducing the surface roughness down to the nanometer range (see Fig. 8).

**Figure 9:**
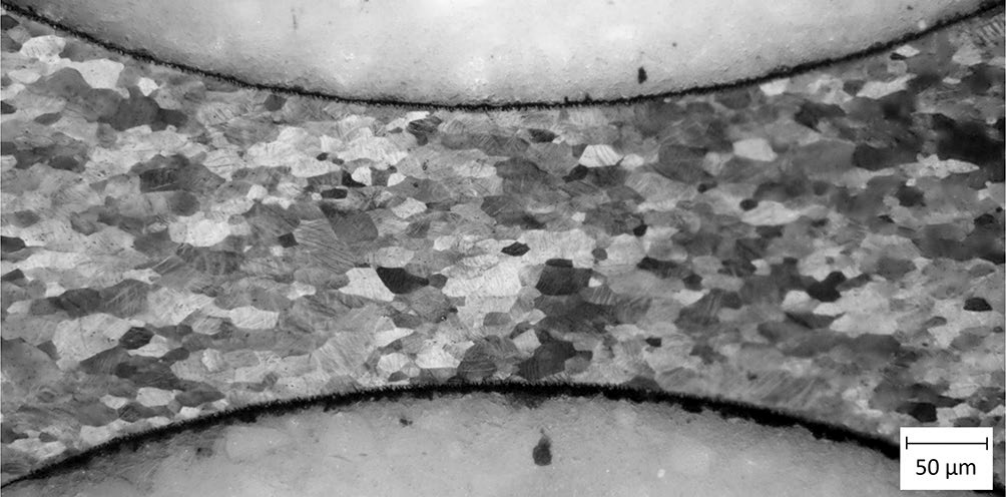
Etched micrograph of the laser-cut Nitinol hinge zone made by femtosecond laser and subsequent electro polishing process, Image kindly provided by Admedes GmbH.

**Figure 10:**
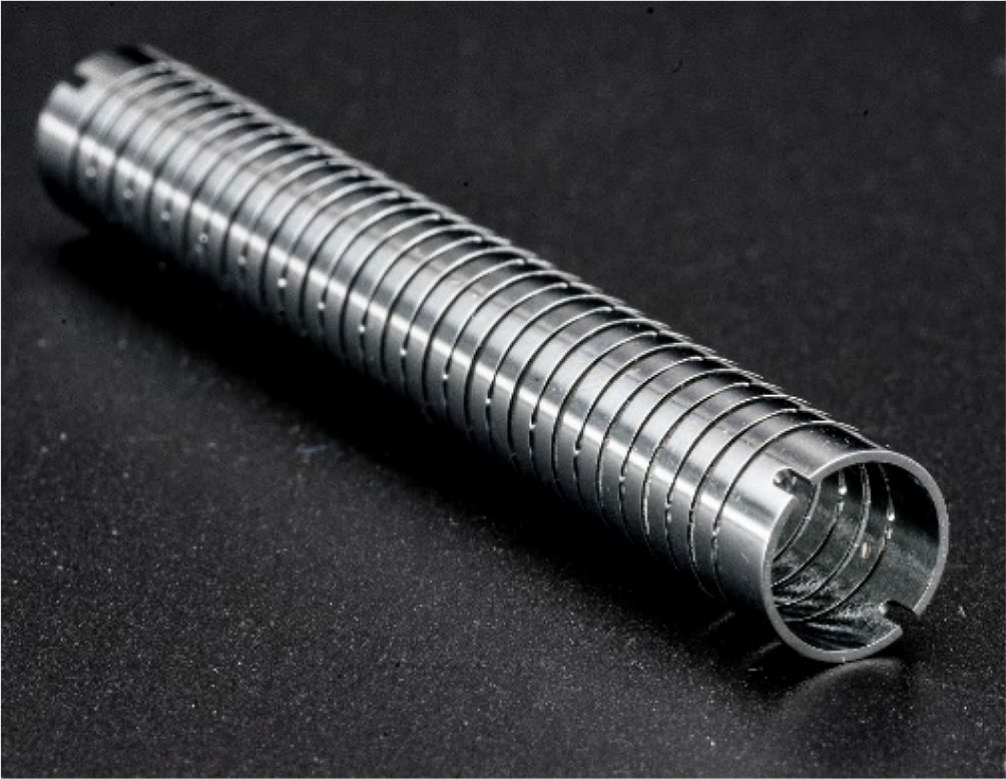
Complete Nitinol monolithic bending section.

The influence of the laser process on the microstructure was investigated using a micrograph (Fig. 9) and no significant change of the microstructure due to laser processing in proximity to the cutting region was found. The manufactured bending section is shown in Fig. 10.

### Experimental testing of monolithic Nitinol bending section

**Figure 11:**
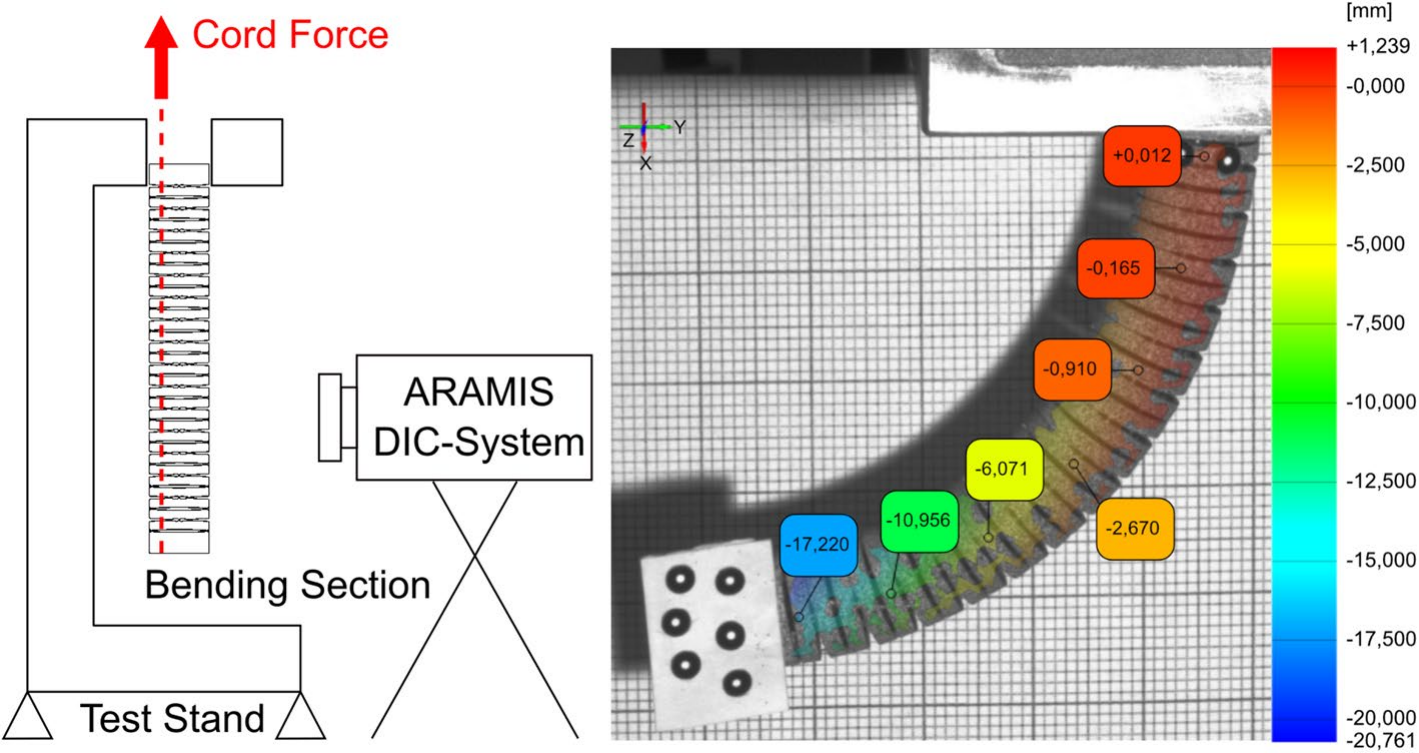
Principle sketch of the test setup to determine the necessary actuating forces, the bending line and the local deformation in the joint area (left)—displacement of the bending section in vertical direction determined by GOM ARAMIS software superimposed on one camera image of the DIC system (right).

The experimental testing of the bending section was performed on a ZwickRoell tensile test rig. A mounting adapter was developed to attach and actuate the bending section (Fig. 11). In order to measure the forces of the pull wires, thin cords were attached to the tip of the bending section guided through the inner lumen and clamped on the upper traverse of the tensile test machine. To avoid additional bending resistance of the system. Flexible cords were used instead of stiff bowden cables in these experiments. For the measurement of the actuation force a 10 N load cell was used. The movement of the bending section was observed using an 4 Megapixel GOM® ARAMIS 3D Digital image correlation (DIC) deformation measurement system. A sketch of the test setup is shown in Fig. 11.

**Figure 12:**
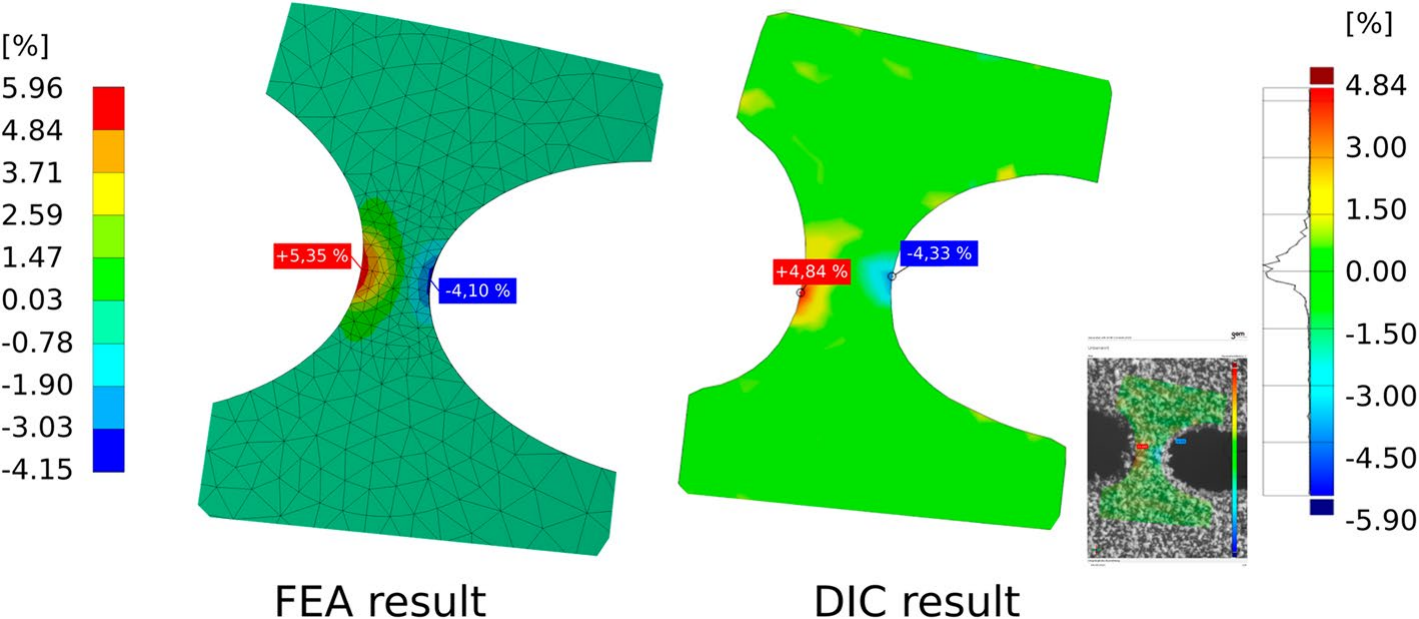
Comparison of numerical (left, FEA result) and experimentally (right, 2D DIC data) determined major strain in the hinge zone at full joint deflection.

The bending line of the bending section was determined using marker points applied to the tip of the bending section in addition to the gray scale pattern. Figure 11 examplarily shows the displacement of the bending section at almost full deflection in vertical (x) direction determined by GOM® ARAMIS software superimposed on one camera image of the DIC system. The measured activation force corresponded to the value determined by means of FEM of 0.82 N. Since the spatial resolution of the 3D DIC data system used was not sufficient to resolve the local strain in the joint areas, additionally a 2D DIC system (GOM® ARAMIS Software, Nikon D800 with Tokina AF 100 mm f/2.8 AT-X Pro D macro lens) was used to determine the local deformation in the compliant hinge zone. In Fig. 12 the comparison of numerically and experimentally determined major strain in the joint area at full hinge deflection is shown.

The numerically and experimentally derived strain data show good qualitative and quantitative agreement. The numerically determined maximum value in the tensile direction of 5.65% is higher than the experimentally determined maximum value of 4.84%. However, it should be noted that the DIC software cannot determine the strain perfectly up to the edge of the joint. The maximal tolerable strain of 6% was not exceeded at full joint deflection. At this point it is necessary to mention that these comparatively high strain values are only suitable for low cycle numbers. Although the joint was fully deflected 10 times without damage during the tests carried out, no fatigue tests were performed as part of these investigations.

In addition to investigating the deformation behavior of the bending section, the actuation forces were determined using the same test setup and compared with the simulation data. The required forces of the monolithic Nitinol bending section at 100° hinge deflection are listed in Table 2.

**Table 2:** Actuation Forces at 100° deflection of the bending section.

Construction	Force at 100° Joint deflection
Nitinol monolithic experiment	0.80 N
Nitinol monolithic simulation	0.82 N
Reference design—riveted stainless steel construction with outer mesh	1.6 N

Experimental data shows, that the bending section is fully deflected (100° bending angle) at a force of 0.8 N. Higher loads just lead to further material deformation within the whole construction. The experimentally determined load is in good agreement to the result of the FEA simulation (F = 0.82 N). Reference testing was also carried out with a bending section currently used on the market. It consists of a riveted stainless steel construction additionally surrounded by a stainless steel mesh.

**Figure 13:**
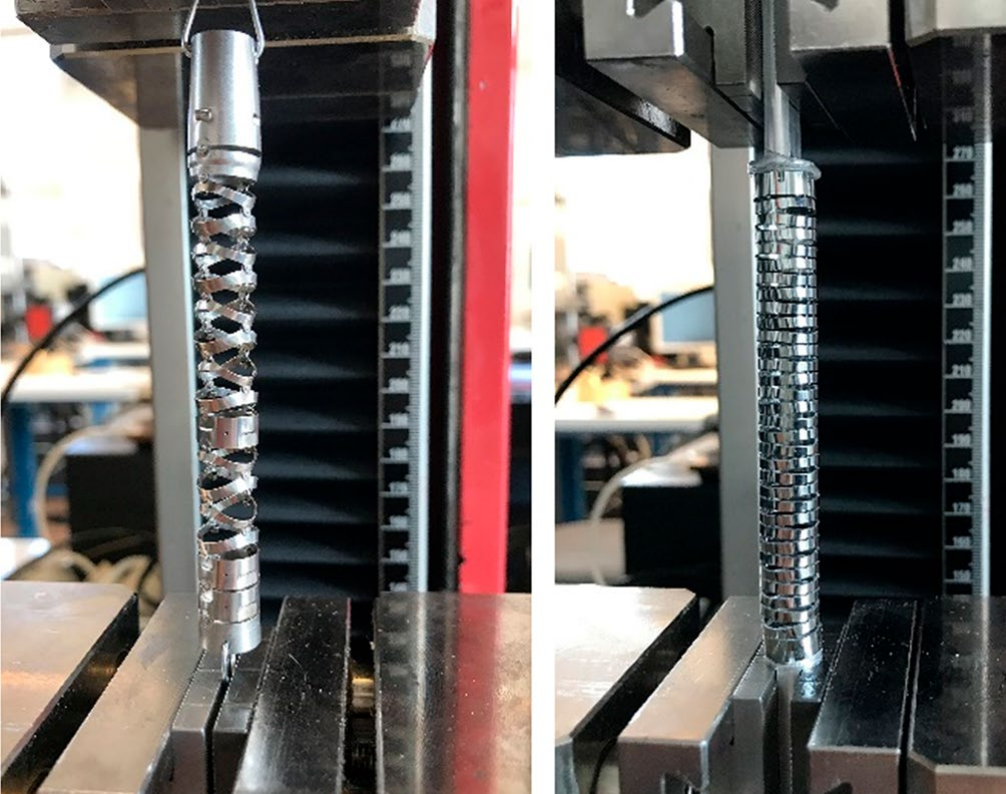
Determination of the failure load under uniaxial tensile loading on a reference bending section made of stainless steel (left image) and the monolithic Nitinol bending section (right image).

This construction has a larger maximum deflection angle. For this test, however, the joint was also only deflected up to an angle of 100° for adequate comparison. The actuating forces are about twice as high compared to the Nitinol bending section. During the tests it was also observed that, in contrast to the Nitinol bending section, the reference joint does not automatically return to the zero position when the force is removed. After the bending tests, both, the Nitinol bending section and the riveted stainless steel construction were tested under uniaxial tensile loading until complete failure and the maximum loads were determined (Fig. 13). The measurement of the failure load was conducted with a calibrated 1 kN load cell.

**Figure 14:**
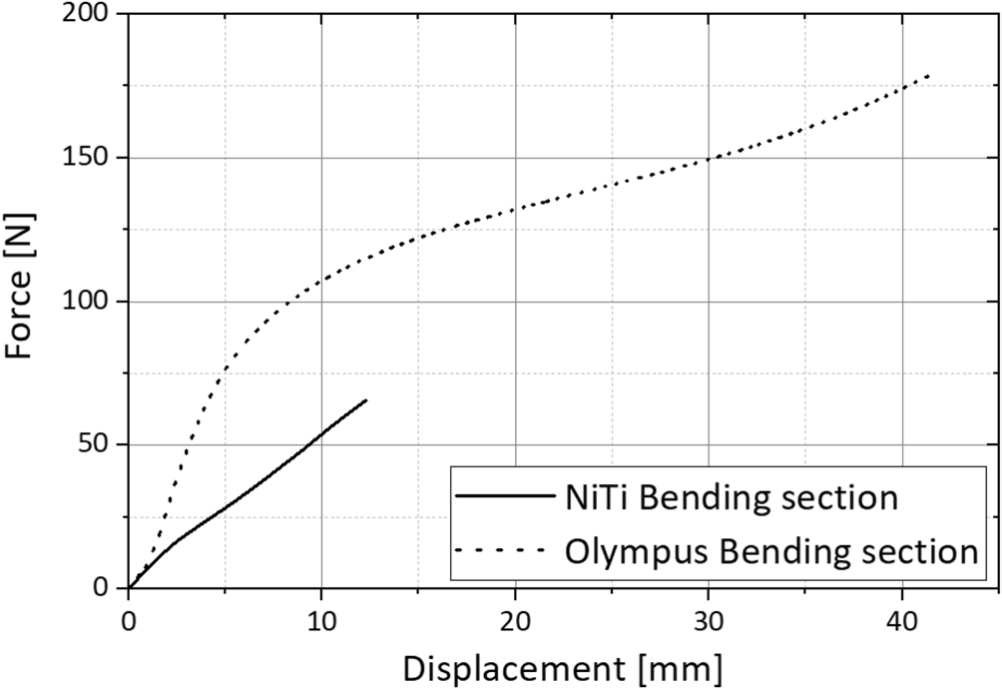
Force–displacement diagram for uniaxial tensile loading of the Nitinol bending section and a reference bending section by Olympus made of stainless steel.

The riveted reference design has a significantly higher failure load of 178 N compared to the monolithic Nitinol design (F_max_ = 66 N, Fig. 14).

Relative to the residual cross-section, the mean stress in the area of the solid joint is 615 N/mm^2^. Although this value is lower than the tensile strength of the material, Fig. 13 clearly shows that the joint areas are additionally stressed in bending, which leads to a locally higher stress. The residual cross section of the riveted reference part is significantly larger in the area of the riveting than in the monolithic Nitinol bending section which is the reason for the higher failure load. On the other hand, the usable inner diameter of the Nitinol component (wall thickness 0.533 mm) is about 10% larger than that of the reference bending section at the rivets Although the uniaxial tensile test certainly does not correspond to possible misuse load cases in real use of a bending section in endoscopes, the test provides valuable information on the load capacity of the design.

## Conclusion

In this paper an approach for a monolithic bending section manufactured from a Nitinol tube using laser cutting technology for the application in single use laparoscopic devices is presented. Compared to a state of the art bending section made of stainless steel, the required actuation force could be reduced by half and the inner diameter of the bending section could be increased approximately by 10%, clearing space for other functional components inside the instrument. Additionally, the monolithic design exhibits a defined center position, shows no axial play and no friction effects during operation. The developed design takes into account an axial preload force, which is induced by the Nitinol actuators to be used. Due to the thereby resulting required height of the cylindrical sections, the developed design cannot achieve bending radius values as small as state of the art riveted bending sections, which, however, are not designed for the use with these types of actuators. This work provides relevant insights into the design of monolithic bending sections for flexible single use instruments.
